# Traumatic rupture of a non-parasitic simple hepatic cyst presenting as an acute surgical abdomen: Case report

**DOI:** 10.1016/j.ijscr.2019.10.051

**Published:** 2019-10-28

**Authors:** Jinyoung Park

**Affiliations:** Trauma Center, Department of Surgery, School of Medicine, Kyungpook National University, Kyungpook National University Hospital, Daegu, South Korea

**Keywords:** Nonparasitic simple hepatic cyst, Rupture, Deroofing

## Abstract

**Introduction:**

Non-parasitic simple hepatic cyst is a very common type of benign liver disease, occurring in approximately 1–5% of the general population. These cysts are usually asymptomatic and are conservatively followed up without treatment. Some of these cysts, however, are associated with complications, including infection, hemorrhage, obstructive jaundice, portal hypertension, and rupture.

**Presentation of case:**

A 74-year-old woman was transferred to our trauma center with epigastric pain after being knocked down by a cultivator. An abdominal computed tomography (CT) scan showed fluid collection and cystic lesion around the caudate lobe of the liver. Operative exploration showed that she had a ruptured hepatic cyst that originated from the caudate lobe of the liver. The cyst was deroofed to the margins of the liver parenchyma, and the internal surfaces of the cyst walls was subjected to electrocoagulator ablation.

**Discussion:**

Open or laparoscopic cyst deroofing is a safe and effective treatment for non-parasitic simple hepatic cysts. Laparoscopic deroofing may also be difficult following rupture of non-parasitic simple hepatic cysts associated with infection or hemorrhage, as these cysts may collapse and the line of resection may be unclear. Laparoscopic ultrasound is a useful adjunct to delineate the boundaries of the cyst.

**Conclusion:**

Traumatic rupture of a non-parasitic simple hepatic cyst is a very rare complication, but can lead to acute abdomen. In patients who are known to have non-parasitic simple hepatic cyst, rupture of cyst could be included in the differential diagnosis of acute abdomen.

## Highlights

• Traumatic rupture of a non-parasitic simple hepatic cyst is very unusual.
• Open or laparoscopic cyst deroofing is a safe and effective treatment for non-parasitic simple hepatic cysts.
• Rupture of a non-parasitic simple hepatic cyst could be included in the differential diagnosis of acute abdomen.

## 1 Introduction

Non-parasitic simple hepatic cyst is a very common type of benign liver disease, occurring in approximately 1–5% of individuals in the general population [[1], [2], [3], [4]]. Recent advances in imaging techniques have resulted in increased incidental detection of these cysts by abdominal ultrasonography or computed tomography (CT) [1,2]. These cysts are usually asymptomatic, allowing conservative follow up without specific treatment [5,6]. Some of these cysts, however, are associated with complications such as infection, hemorrhage, obstructive jaundice, portal hypertension, and rupture [[7], [8], [9], [10], [11]]. There have been few reports describing the rupture of non-parasitic simple hepatic cysts [1,3,[8], [9], [10], [11]]. Therefore, there are no standard treatment guidelines or surgical indications for ruptured non-parasitic simple hepatic cysts. This report describes a patient who experienced traumatic rupture of a non-parasitic simple hepatic cyst and who was successfully treated by deroofing the cyst. This work has been reported in line with the SCARE criteria [12].

## 2 Presentation of case

**Fig. 1:**
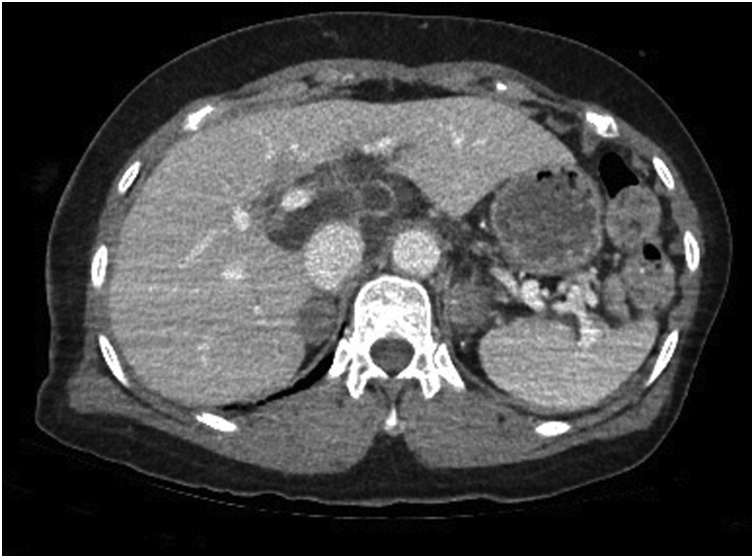
Axial view on an abdominal computed tomography scan of this patient, showing fluid collection and a cystic lesion around the caudate lobe of the liver.

**Fig. 2:**
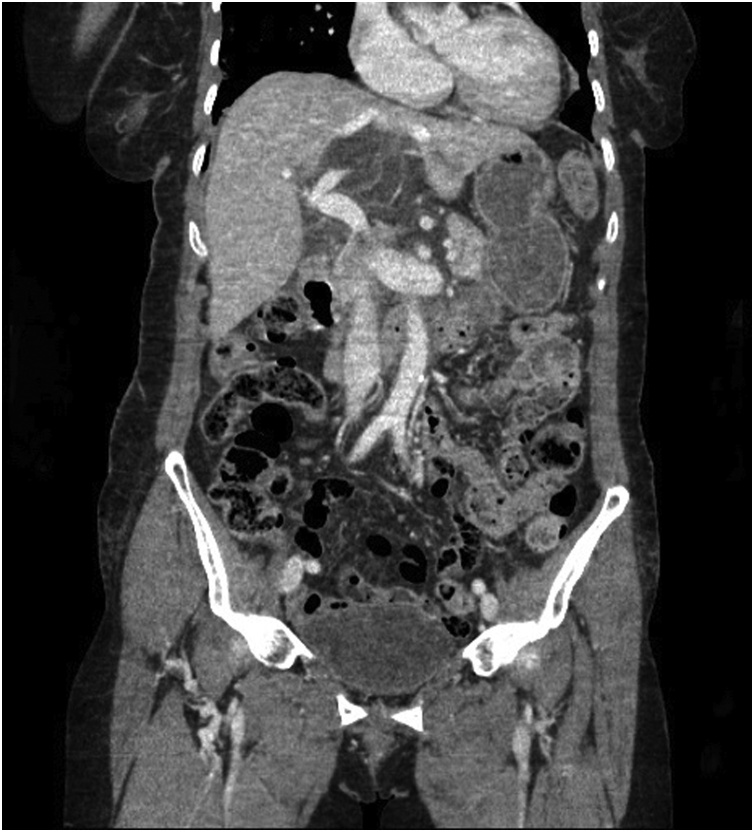
Coronal view on an abdominal computed tomography scan of the patient, showing fluid collection and a cystic lesion around the caudate lobe of the liver.

**Fig. 3:**
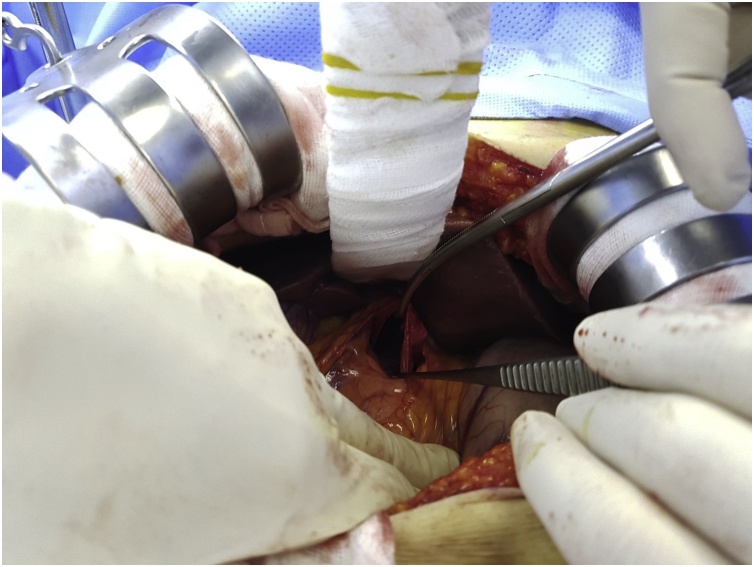
Photograph taken during exploratory laparotomy, showing a ruptured hepatic cyst that had originated from the caudate lobe of the liver.

**Fig. 4:**
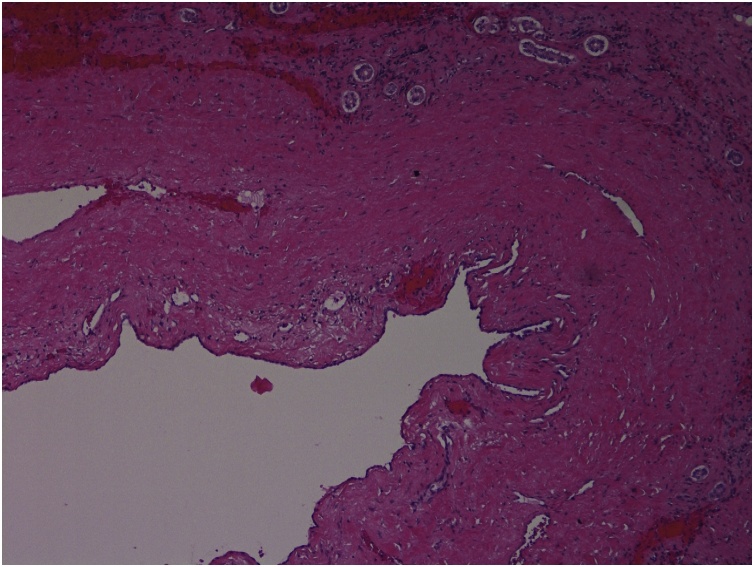
Histological examination of the cyst, showing flat epithelium with fibrous connective tissue (H & E staining, x100 original magnification).

A 74-year-old woman was transferred to our trauma center with epigastric pain after being knocked down by a cultivator. She had been on medication for hypertension and arthritis. Upon admission, she complained of pain in the upper abdomen and both lower chest regions. Her vital signs at admission included a blood pressure of 145/90 mmHg, a heart rate of 86 beats per minute, a respiratory rate of 22 breaths per minute, and a body temperature of 36.5 °C. Physical examination revealed severe rebound tenderness in the epigastrium. Laboratory findings showed that her hemoglobin concentration and white blood cell and platelet counts were within normal ranges. Renal and hepatic function test results were also within normal limits, except for elevated concentrations of aspartate aminotransferase (310 U/L; reference range, <33 U/L) and alanine aminotransferase (181 U/L; reference range, <33 U/L). A chest CT scan showed fractures of several right ribs and bilateral scanty pneumothorax. An abdominal CT scan showed fluid collection and cystic lesion around the caudate lobe of the liver (Fig. 1, Fig. 2). Operative exploration showed that she had a ruptured hepatic cyst that originated from the caudate lobe of the liver. The perforation site was located on the anterior side of the cyst (Fig. 3). Some fluid of a serous nature had collected in the lesser sac. The cyst was deroofed to the margins of the liver parenchyma, and the internal surfaces of the cyst walls were carefully searched to determine the site of biliary communication. Because no biliary communication was visible, the remaining epithelium in the inner wall was subjected to electrocoagulator ablation. Retrospective questioning of the patient revealed that she had been diagnosed with an hepatic cyst several years earlier, and that the cyst had since been observed without any treatment. Pathologic examination showed a non-parasitic simple hepatic cyst (Fig. 4). Her postoperative course was uneventful, and she was discharged from the hospital 10 days after surgery. She remained asymptomatic at follow-up 3 months after discharge.

## 3 Discussion

Non-parasitic simple hepatic cysts are a common type of benign liver disease, occurring in approximately 1–5% of the general population [1,3]. Increased utilization of diagnostic imaging methods, such as ultrasonography and CT, has enabled the identification of increasing numbers of hepatic cysts in the general population [2]. These cysts are found more frequently in women than in men, at a ratio of 3:1 [4,6]. Although they are usually asymptomatic, they can produce symptoms, depending on their size, anatomic location, or presence of complications. Most commonly, cyst enlargement can induce a foreign body sensation, epigastric pain, nausea, vomiting and/or postprandial bloating. Non-parasitic simple hepatic cysts are often associated with various complications, including obstructive jaundice, portal hypertension, inferior vena cava thrombosis, and acute pulmonary embolism. Although rare, these complications can develop due to infection, torsion, intracystic hemorrhage, or rupture of the cyst into the peritoneal cavity, biliary tree or adjacent hollow viscus such as the colon. In general, ruptures of parasitic hepatic cysts, also called hydatid cyst ruptures, tend to be caused by *Echinococcus* species and are complications frequently associated with these cysts [5]. In contrast, ruptures of non-parasitic simple hepatic cysts are rare and can be spontaneous or caused by infection, trauma, or iatrogenic injury [1]. Only 21 English publications that describe ruptures of non-parasitic simple hepatic cysts were published in PubMed from 1974 to 2019 [1,[3], [4], [5], [6], [7], [8], [9], [10], [11],[13], [14], [15], [16], [17], [18], [19], [20], [21], [22], [23]] (Table 1). Traumatic rupture of a non-parasitic simple hepatic cyst, as occurred in our patient, is very unusual.

**Table 1:** Literature review of ruptured non-parasitic simple hepatic cysts.

Reference	Sex	Age (years)	Cyst size (cm)	Location	Cause of rupture	Treatment	Outcome
Inoue et al. [1]	F	59	10	Left lobe	Spontaneous	Open cyst fenestration, omental transposition	Uneventful
Shimada et al. [3]	F	61	?	Right lobe	Spontaneous	Laparoscopic deroofing	Uneventful
Marques et al. [4]	M	48	9	Right lobe	Spontaneous	Laparoscopic deroofing	Uneventful
Imaoka et al. [5]	F	67	10.5	Right lobe	Spontaneous	Laparoscopic deroofing	Uneventful
Miliadis et al. [6]	M	70	13	Right lobe	Spontaneous	Open deroofing, omentoplasty	Uneventful
Hotta et al. [7]	F	62	13	Right lobe	Spontaneous	Percutaneous aspiration, injection of minocycline hydrochloride	Uneventful
Simon et al. [8]	M	63	?	Right lobe	?	Conservative treatment	Uneventful
Vannucchi et al. [9]	M	73	?	Right lobe	?	Surgical treatment	?
Cheung et al. [10]	F	73	17	Right lobe	Spontaneous	Laparoscopic unroofing	Uneventful
Salemis et al. [11]	M	50	17	Left lobe	Spontaneous	Open unroofing	Uneventful
Marion et al. [13]	F	37	18	Right lobe	?	Open cystectomy	Uneventful
Ueda et al. [14]	F	64	10	Right lobe	Spontaneous	Percutaneous aspiration, injection of minocycline hydrochloride	Uneventful
Shutsha et al. [15]	F	67	?	Multiple	Coughing fit	Conservative treatment	Uneventful
Kanazawa et al. [16]	M	78	?	Right lobe	Spontaneous	Intracystic ethanol injection	Uneventful
Ishikawa et al. [17]	F	42	10	Segment 4 & 5	Spontaneous	TAE, Open cystectomy	Uneventful
Carles and van [18]	M	76	19	Right lobe	Anticoagulation	Omentum placed over the ruptured cyst	Death
Yamaguchi et al. [19]	M	61	13	Left lobe	Spontaneous	Left trisegmentectomy	Uneventful
Payatakes et al. [20]	?	62	9.5	Right lobe	?	Partial excision, external drainage	Uneventful
Akriviadis et al. [21]	F	48	?	Left lobe	Spontaneous	Conservative treatment	Uneventful
Ayyash and Haddad [22]	F	36	4	Right lobe	Spontaneous	Cyst excision	Uneventful
Brunes [23]	F	54	25	Left lobe	Spontaneous	Partial excision of cyst	Uneventful

M; male, F; female, TAE; Transcatheter arterial embolization.

Because they can regress spontaneously, especially when ranging from 2 to 4 cm in diameter, asymptomatic non-parasitic simple hepatic cysts require no treatment. Cysts larger than 4 cm in diameter should be monitored by repeated imaging; however, if the cyst remains unchanged for 2 years, monitoring may be stopped [6].

Non-parasitic simple hepatic cysts have been treated by various methods, including percutaneous needle aspiration and open or laparoscopic surgery, with varying degrees of success. Ultrasound- or CT-guided percutaneous needle aspiration is safe and relatively noninvasive and can also identify the contents of the cyst. This method can therefore be recommended as first-line treatment for patients with high surgical risk or polycystic liver disease. Percutaneous needle aspiration, however, should be considered only after eliminating the possibility of a malignant or infectious etiology or a cystobiliary communication. Although this treatment is associated with a high (>80%) rate of recurrence, the likelihood of recurrence can be reduced by about 20% when percutaneous needle aspiration is combined with injection of a sclerosing agent, such as minocycline hydrochloride or tetracycline chloride, as these agents promote coagulation-induced necrosis of the cyst epithelium and effectively obliterate cysts [1,7].

Open or laparoscopic cyst deroofing is a safe and effective treatment for non-parasitic simple hepatic cysts. Recurrence rates can also be reduced by combining deroofing with argon beam coagulation or electrocoagulation, methods that destroy the remaining epithelium. Moreover, placement of an omental transposition flap after deroofing can reduce recurrence rates. Laparoscopic management has been shown to be a new, less-invasive therapeutic option [[2], [3], [4], [5]]. Compared with open deroofing, laparoscopic deroofing is associated with shorter hospital stay, more rapid return to normal activities, and lower morbidity rates. The feasibility of laparoscopic treatment depends on the location and size of the cysts. Cysts situated in the superior and posterior segments of the liver are more difficult to approach, thus requiring different port positions and additional ports [10]. Open deroofing is preferable, however, for very large hepatic cysts and cysts located at laparoscopically inaccessible sites, despite open deroofing being associated with higher morbidity rates. Laparoscopic complete excision of cysts located in the superior and posterior segments of the liver or deep within hepatic parenchyma may not be possible. Laparoscopic deroofing may also be difficult following rupture of non-parasitic simple hepatic cysts associated with infection or hemorrhage, as these cysts may collapse and the line of resection may be unclear [5]. Laparoscopic deroofing may have been possible in light of operative findings in our patient. However, since we did not know that the patient had non-parasitic simple hepatic cyst preoperatively, open deroofing was performed. Laparoscopic ultrasound is a useful adjunct to delineate the boundaries of the cyst [10]. Cystobiliary communications can be identified by intraoperative bile leak tests using agents such as indigo carmine and indocyanine green [3].

Roux-en-Y internal drainage with cystojejunostomy has been proposed as treatment for cysts communicating with the bile duct. However, this method could lead to complications, such as cholangitis and sepsis, which require repeated postoperative antibiotic treatments. More radical approaches, including complete cyst excision and partial hepatectomy, have been recommended if the possibility of malignancy cannot be completely ruled out, but these approaches carry significantly higher morbidity rates [5]. These highly invasive approaches are poorly tolerated by patients at high surgical risk and are almost unacceptable for patients presenting with benign diseases, despite the reported recurrence rate being 0% [1].

Recurrence rates of hepatic cyst after open and laparoscopic surgery vary. Some authors reported a higher rate of recurrence after laparoscopic surgery. While others concluded that the recurrence rates after laparoscopic surgery were acceptable and comparable to those of conventional open surgery [2,24].

## 4 Conclusion

In conclusion, traumatic rupture of a non-parasitic simple hepatic cyst is a very rare complication, but can lead to acute abdomen. In patients who are known to have non-parasitic simple hepatic cyst, rupture of cyst could be included in the differential diagnosis of acute abdomen.

## Funding

None. No funding or grant support.

## Ethical approval

Because this was a report of an interesting case, and not atrial or observational research, we had an exemption from ethical approval.

## Consent

Written informed consent was obtained from the patient for publication of this case report and accompanying image.

## Author contribution

Jinyoung Park was involved with the case and writing of the manuscript, operation and general management of the patient and revised the manuscript for important intellectual content.

## Registration of research studies

None.

## Guarantor

Jinyoung Park.

## Provenance and peer review

Not commissioned, externally peer-reviewed.

## Declaration of Competing Interest

None.

## References

[1] Inoue K., Iguchi T., Ito S., Ohga T., Nozoe T., Shirabe K., Ezaki T., Maehara Y. (2015). Rerupture of nonparasitic liver cyst treated with cyst fenestration: a case report. Surg. Case Rep..

[2] Antonacci N., Ricci C., Taffurelli G., Casadei R., Minni F. (2014). Systematic review of laparoscopic versus open surgery in the treatment of non-parasitic liver cysts. Updates Surg..

[3] Shimada S., Hara Y., Wada N., Nakahara K., Takayanagi D., Ishiyama Y., Maeda C., Mukai S., Sawada N., Yamaguchi N., Sato Y., Hidaka E., Ishida F., Kudo S.E. (2016). Spontaneously ruptured hepatic cyst treated with laparoscopic deroofing and cystobiliary communication closure: a case report. Asian J. Endosc. Surg..

[4] Marques A., Camarneiro R., Silva R., Rodrigues A., Dionísio I., Ferreira Á, Brito E., Melo M. (2019). Laparoscopic deroofing of a ruptured hepatic cyst presenting as an acute abdomen. J. Surg. Case Rep..

[5] Imaoka Y., Ohira M., Kobayashi T., Shimizu S., Tahara H., Kuroda S., Ide K., Ishiyama K., Ohdan H. (2016). Elective laparoscopic deroofing to treat the spontaneous rupture of a large simple liver cyst: a case report. Surg. Case Rep..

[6] Miliadis L., Giannakopoulos T., Boutsikos G., Terzis I., Kyriazanos I.D. (2010). Spontaneous rupture of a large non-parasitic liver cyst: a case report. J. Med. Case Rep..

[7] Hotta M., Yoshida H., Makino H., Yokoyama T., Maruyama H., Uchida E. (2015). Spontaneous rupture of a simple hepatic cyst: report of a case. J. Nippon Med. Sch..

[8] Simon T., Bakker I.S., Penninga L., Nellensteijn D.R. (2015). Haemorrhagic rupture of hepatic simple cysts. BMJ Case Rep..

[9] Vannucchi A., Masi A., Vestrini G., Tonelli F. (2016). Extraperitoneal hemorrhagic rupture of a simple hepatic cyst. A case report and literature review. Ann. Ital. Chir..

[10] Cheung F.K., Lee K.F., John W., Lai P.B. (2005). Emergency laparoscopic unroofing of a ruptured hepatic cyst. JSLS.

[11] Salemis N.S., Georgoulis E., Gourgiotis S., Tsohataridis E. (2007). Spontaneous rupture of a giant non parasitic hepatic cyst presenting as an acute surgical abdomen. Ann. Hepatol..

[12] Agha R.A., Borrelli M.R., Farwana R., Koshy K., Fowler A.J., Orgill D.P. (2018). SCARE group. The SCARE 2018 statement: updating consensus Surgical CAse REport (SCARE) guidelines. Int. J. Surg..

[13] Marion Y., Brevartt C., Plard L., Chiche L. (2013). Hemorrhagic liver cyst rupture: an unusual life-threatening complication of hepatic cyst and literature review. Ann. Hepatol..

[14] Ueda J., Yoshida H., Taniai N., Mineta S., Kawano Y., Uchida E. (2010). A case of spontaneous rupture of a simple hepatic cyst. J. Nippon Med. Sch..

[15] Shutsha E., Brenard R. (2003). Hepatic cyst rupture after a coughing fit. J. Hepatol..

[16] Kanazawa A., Yoshioka Y., Inoi O., Kubo S., Kinoshita H. (2003). Intracystic hemorrhage with spontaneous rupture of liver cyst complicated by infection: a case report. Osaka City Med. J..

[17] Ishikawa H., Uchida S., Yokokura Y., Iwasaki Y., Horiuchi H., Hiraki M., Kinoshita H., Shirouzu K. (2002). Nonparasitic solitary huge liver cysts causing intracystic hemorrhage or obstructive jaundice. J. Hepatobiliary. Surg..

[18] Carels R.A., van Bommel E.F. (2002). Ruptured giant liver cyst: a rare cause of acute abdomen in a haemodialysis patient with autosomal dominant polycystic kidney disease. Neth. J. Med..

[19] Yamaguchi M., Kuzume M., Matsumoto T., Matsumiya A., Nakano H., Kumada K. (1999). Spontaneous rupture of a nonparasitic liver cyst complicated by intracystic hemorrhage. J. Gastroenterol..

[20] Payatakes A.H., Kakkos S.K., Solomou E.G., Tepetes K.N., Karavias D.D. (1999). Surgical treatment of non-parasitic hepatic cysts: report of 12 cases. Eur. J. Surg..

[21] Akriviadis E.A., Steindel H., Ralls P., Redeker A.G. (1989). Spontaneous rupture of nonparasitic cyst of the liver. Gastroenterology.

[22] Ayyash K., Haddad J. (1988). Spontaneous rupture of a solitary nonparasitic cyst of the liver. Case report. Acta Chir. Scand..

[23] Brunes L. (1974). Rupture of a solitary nonparasitic cyst of the liver. Report of a case. Acta Chir. Scand..

[24] Gigot J.F., Legrand M., Hubens G., de Canniere L., Wibin E., Deweer F., Druart M.L., Bertrand C., Devriendt H., Droissart R., Tugilimana M., Hauters P., Vereecken L. (1996). Laparoscopic treatment of nonparasitic liver cysts: adequate selection of patients and surgical technique. World J. Surg..

